# A Possible Primordial Acetyleno/Carboxydotrophic Core Metabolism

**DOI:** 10.3390/life10040035

**Published:** 2020-04-07

**Authors:** Jessica Sobotta, Thomas Geisberger, Carolin Moosmann, Christopher M. Scheidler, Wolfgang Eisenreich, Günter Wächtershäuser, Claudia Huber

**Affiliations:** 1 Lehrstuhl für Biochemie, Department Chemie, Technische Universität München, Lichtenbergstraße 4, 85748 Garching, Germany; Jessy.Sobotta@web.de (J.S.); thomas.geisberger@tum.de (T.G.); carolin.moosmann@tum.de (C.M.); christopher.scheidler@cup.lmu.de (C.M.S.); wolfgang.eisenreich@mytum.de (W.E.); 2 209 Mill Race Drive, Chapel Hill, NC 27514, USA; gwmunich@bellsouth.net

**Keywords:** origin of life, chemical evolution, early metabolism, transition metal catalysis, carbon fixation, nickel sulfide, acetylene, carbon monoxide

## Abstract

Carbon fixation, in addition to the evolution of metabolism, is a main requirement for the evolution of life. Here, we report a one-pot carbon fixation of acetylene (C_2_H_2_) and carbon monoxide (CO) by aqueous nickel sulfide (NiS) under hydrothermal (>100 °C) conditions. A slurry of precipitated NiS converts acetylene and carbon monoxide into a set of C_2–4_-products that are surprisingly representative for C_2–4_-segments of all four central CO_2_-fixation cycles of the domains Bacteria and Archaea, whereby some of the products engage in the same interconversions, as seen in the central CO_2_-fixation cycles. The results suggest a primordial, chemically predetermined, non-cyclic acetyleno/carboxydotrophic core metabolism. This metabolism is based on aqueous organo–metal chemistry, from which the extant central CO_2_-fixation cycles based on thioester chemistry would have evolved by piecemeal modifications.

## 1. Introduction

All scientific theories concerning the origin and early evolution of life have to consider carbon fixation and the evolution of metabolism. Extant carbon fixation cycles are seen as successors of primordial carbon fixation, and their evolutionary history has been reconstructed as a “phylometabolic” tree [1]. The extant biosphere mainly owes its existence to CO_2_-fixation. Scientific theories concerning the origin and early evolution of life are expected to be explanatory for this overarching fact. However, any attempt to project from extant CO_2_-fixation back to a primitive CO_2_-based core metabolism as wellspring for all biosynthetic pathways faces severe chemical hurdles. Due to its high chemical stability, the conversion of CO_2_ into core metabolic constituents mainly requires energy coupling by phosphorylation and thioester formation, as well as a nucleophilic attack by carbanion intermediates, and all that is aggravated by the number of C_1_-extensions. Despite recent findings of acetate and pyruvate formation from CO_2_ through inorganic catalysis [2,3], alternative geochemically-available carbon sources should be considered. We chose acetylene and CO as primordial carbon nutrients with the following benefits: (a) availability in volcanic-hydrothermal settings [4,5,6,7]; (b) high chemical reactivity with the avoidance of energy coupling; (c) low C-oxidation numbers; (d) CO also serving as reducing agent; (e) strong ligation to catalytic transition metal centers, notably of Ni(Fe)S; (f) propensity to engage in organo–nickel reactions instead of carbanion condensations, (g) acyl-nickel activation instead of thioester activation; and (h) C_2_-extensions by acetylene ligands instead of C_1_-extensions by CO_2_, with the consequence of a lessened number of required reaction steps. Our findings may be seen as a hint to the evolution of extant carbon fixation cycles through the suggestion of replacing them through a linear reaction system with the inherent possibility of evolving cyclic reaction systems.

## 2. Materials and Methods

All chemicals were purchased from Sigma Aldrich GmbH (D-Steinheim) in the highest purity available. Acetylene was purchased from Linde AG (D-Pullach), carbon monoxide 2.5 and argon 4.6 were purchased from Westfalen AG (D-Münster), and ^13^CO was purchased from Cambridge Isotopes Laboratories Inc. (Tewksbury, MA, USA).

In a typical run, a 125 mL glass serum bottle was charged with 0.5 or 1.0 mmol NiSO_4_ • 6H_2_O and closed with a silicon stopper. Additionally, 0.5 mmol β-Ni(OH)_2_ or 0.5 mmol FeSO_4_ • 7 H_2_O was charged in run B or D (Table 1), respectively. To achieve a constant ion strength, run B was supplemented with 0.5 mmol Na_2_SO_4_. The bottle was evacuated three times and filled with argon, finally ending in a deaerated state. Subsequently, the bottle was charged with argon-saturated water (calculated for a final volume of 5 mL), with 0.5 or 1.0 mL argon-saturated 1M Na_2_S solution, with 0.5 mL 1M NaOH solution, and finally with 60 mL of CO and 60 mL of acetylene, using gas-tight syringes for injection. For consecutive reactions, the conditions of run A (see Table 1) were applied, replacing acetylene by 0.5 mmol of the indicated substrates and 60 mL of CO. To confirm the authenticity of the products, ^13^CO or D_2_O were used in otherwise identical experiments. Reactions were carried out at 105 °C. After 7 days, the reaction mixture was allowed to cool down and was centrifuged at 10,000 rpm for 5 minutes. The pH was measured by a glass electrode, and 1 ml of the supernatant was freeze-dried.

For analysis by gas chromatography-mass spectrometry (GC–MS), the residue was dissolved in 250 μL of anhydrous acetonitrile and derivatized with 250 μL of *N*-tert-butyldimethylsilyl-*N*-methyltrifluoroacetamide (MTBSTFA) for 30 minutes at 70 °C. For the detection of pyruvate, another ml was freeze dried, and the residue was shaken at 40 °C for 90 min in 250 µL of pyridine containing 5 mg of *O*-methylhydroxylamine hydrochloride. Afterwards, 250 µl of MTBSTFA were added, and the solution was kept at 70 °C for 30 min. The analysis of the silylated products was performed with GC–MS using GC-2010, coupled with MS-QP2010, Plus (Shimadzu GmbH, D-Duisburg) with a 30 m × 0.25 mm × 0.25 μm fused silica capillary column (Equity TM5, Supelco, USA-PA-Bellefonte) and an AOC-20i auto injector. Temperature program and settings:

Program 1 (used for mono silylated products): 0–6 min at 60 °C; 6–25 min at 60–280 °C, 10 °C/min; 25–28 min at 280 °C; injector temperature: 260 °C; detector temperature: 260 °C; column flow rate: 1 mL/min; scan interval: 0.5 sec; and injection volume 0.2 μL.

Program 2 (used for multiple silylated products): 0–6 min at 90 °C; 6–25 min at 90–280 °C, 10 °C/min; and 25–28 min at 280 °C. Otherwise, identical to program 1, with an injection volume of 1 μL. Peak assignment was achieved by a comparison of the retention times and mass spectra of purchased reference compounds, as well as data from the National Institute of Standards and Technology (NIST) spectra library. Quantification was performed by external calibration using known concentrations of commercially-available reference compounds.

In additional experiments for the formation of the thioacetic acid S-methyl ester (methyl thioacetate), a 125 ml serum bottle was charged with 2.0 mmol NiSO_4_ • 6H_2_O, closed with a silicon stopper, and deaerated as described above. Subsequently, 1.5 ml of 1M Na_2_S, 0.6 mL of 1M NaOH, 7.9 mL of H_2_O, 25 mL of CH_3_SH, 90 mL of HC≡CH (Table S2 run A), or 45 mL of HC≡CH plus 45 mL of CO (Table S2 run B) were added. Additional runs were performed with ^13^CO or deuterated educts. Reactions were carried out at 105 °C. After one day, the reaction mixture was allowed to cool down. For the isolation of methyl thioacetate, 8 ml of the reaction mixture were extracted with 3 mL of ethyl acetate. The organic phase was dried over Na_2_SO_4_ and analyzed with GC–MS as described above, using an initial oven temperature of 40 °C. The injection volume was 1 µL. Methyl thioacetate showed a retention time of 2.9 min.

## 3. Results

We reacted acetylene and carbon monoxide under fastidiously anaerobic, aqueous conditions at the hydrothermally plausible temperature of 105 °C in the presence of NiS, which was precipitated in situ from Ni_2_SO_4_ with Na_2_S, in the presence or absence β-Ni(OH)_2_. We obtained highly functionalized C_2_–C_4_ products, which are typical for extant carbon fixation cycles (Table 1). Runs with ^13^CO and D_2_O ascertained that the products were genuine reaction products [8]. For these and further quantified products, GC–MS fragmentations and their isotopic labelling indices are listed in Table S1.

The detected C_2_-products acetate and glycolate did not show the ^13^C-label in runs with ^13^CO and therefore must have been the products of acetylene as the sole carbon source undergoing oxidative addition reactions. Ni^2+^ ions may have served as the required oxidant, as evidenced by Ni^0^ particles that have previously been shown to form from NiS with CO as reductants under similar conditions [9]. In agreement with a previous proposal [10], the thioacetic acid S-methyl ester (methyl thioacetate) was formed (2µM in one day) by the reaction of acetylene with methanethiol. A shorter reaction time was chosen due to the chemical instability of methyl thioacetate, which is readily hydrolyzed into acetic acid. Under the chosen conditions, CO did not operate as carbon source (Figure S1), but it enhanced the conversion of acetylene into methyl thioacetate, perhaps by ligand effects (Table 2). Methyl thioacetate can be seen as precursor of acetyl-CoA, which is formed by the reductive acetyl-CoA pathway in extant organisms [11]. In Figure S2, the extant acetyl-CoA pathway is compared to the here-described acetylene reaction. In earlier experiments, methyl thioacetate was found to form by the reaction of CO with methanethiol by Ni(Fe)S catalysis [12]. Methanethiol has been shown to form from CO with Ni(Fe)S/H_2_S [12] or from CO_2_ with FeS/H_2_S [13].

**Figure 1:**
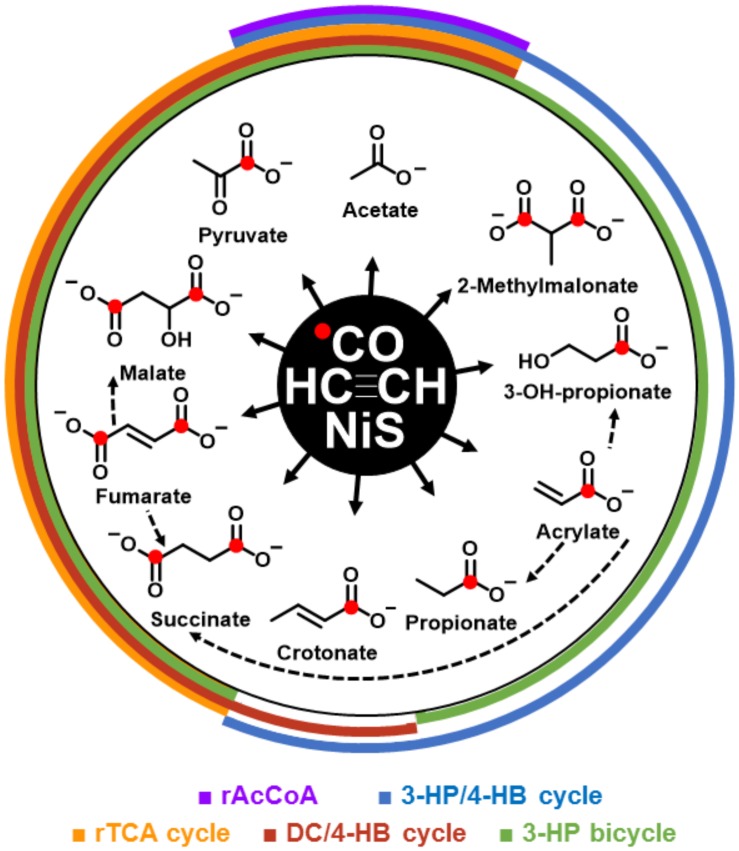
Acetyleno/carboxydotrophic reaction network. NiS-catalyzed reaction network starting from acetylene and carbon monoxide. Observed products are shown with their chemical formula and names; red dots indicate the observed ^13^C label from ^13^CO. Colored semi cycles signify the corresponding parts of the indicated carbon fixation pathways. (rAcCoA: reductive acetyl-CoA pathway; 3-HP/4-HB cycle: 3-hydroxpropinate/4-hydroxybutyrate cycle; rTCA: reductive tricarboxylic acid cycle; DC/4-HB cycle: dicarboxylate/4-hydroxbutyrate cycle; and 3-HP bicycle: 3-hydroxypropionate bicycle. Dotted arrows show observed interconversions between products of the co-radiating, linear pathways.

The formation of the detected organic >C_2_-products required, not only acetylene but also CO as carbon source, as evidenced by the ^13^C-labelling (Table S1). If NiS was precipitated in the presence of β-Ni(OH)_2_, productivity increased significantly, notably from 20 to 32 mM for (acrylate and propionate) or from 4.4 to 5.7 mM for (fumarate, maleate, and succinate) (Table 1, runs A vs. B). The use of β-Ni(OH)_2_ alone (Table 1, run C) or a mixed use of NiS/FeS (Table 1, run D) showed only minor product formation. The organic >C_2_-products had the proper functional groups (COOH, CH=CH, CO, and CHOH) required for core metabolites, from which metabolic pathways could emanate. We detected a set of C_2_–C_4_ products (acetate, pyruvate, propionate, 3-hydroxy propionate, acrylate, malate, fumarate/maleate, succinate, crotonate, and methyl malonate) that were representative of the (hydrolyzed) constituents of the C_2_–C_4_-segments of the four extant central CO_2_-fixation cycles of the domains Bacteria and Archaea. In Figure 1, the observed molecules are shown as co-radiating NiS-catalyzed products from acetylene and carbon monoxide, including possible interconversions in the same system. Through overlapping semicircles, these products are assigned to extant pathways. Figures S3–S6 show, in detail, the known carbon fixation cycles in which products from our abiotic system are highlighted by red boxes: the reductive tricarboxylic acid (rTCA) cycle [14] (Figure S3), the 3-hydroxypropionate–4-hydroxybutyrate (3HP–4HB) cycle [15] (Figure S4), the dicarboxylate-4–hydroxybutyrate (DC–4HB) cycle [16] (Figure S5), and the 3-hydroxypropionate (3HP) bicycle [17] (Figure S6). The rTCA cycle has been recognized as being autocatalytic for acetyl-CoA production [18]. The other three CO_2_-fixation cycles are similarly autocatalytic for acetyl-CoA production and have been recognized for their importance in the evolution of metabolism [19,20,21]. Remarkably, we also found glycerate and (E)-2-methylbut-2-enoate as entry gates into carbohydrate and isoprenoid metabolisms (Table 1). In total, the here-described C_2_–C_5_ products summed up to a concentration of 43 mM (run B; Table 1) in the 5 ml setup, which corresponded to about 10% yield based on acetylene.

Concerning the question of the experimental interconversion of cycle constituents [22,23,24], we performed experiments with the replacement of acetylene by acrylate, fumarate, malate, and succinate as starting materials under otherwise identical conditions. The reaction products of fumarate were succinate, malate and maleate. Acrylate reacted to propionate, β-lactate, succinate, fumarate, and malate. Malate formed maleate, succinate, and fumarate. Succinate remained mainly unchanged and showed only minor conversion to fumarate and malate (Table 3).

## 4. Discussion

The chosen reaction conditions (starting materials, catalysts and reaction parameters) are compatible with a variety of scenarios. They fit particularly well to submarine or terrestrial volcanic-hydrothermal flow scenarios with late Hadean or early Archaean geochemistry. Acetylene is formed by simulating underwater volcanic activities [4], it is found in fumaroles [5] and on solar planets [6], and it is generated by the hydrolysis of calcium carbide (CaC_2_), which, in turn, is formed by the magmatic reaction of calcium oxide with graphite [7]. Biochemically speaking, acetylene is mainly known as an inhibitor for enzymatic reactions [25] and as a substrate for acetylene hydratase, an FeS enzyme with a tungstopterin cofactor that functions biosynthetically [26], or as detoxifying enzyme [27], and it may be more widespread than previously suspected [28]. Early on, when acetylene abundance would have been greater than today, a precursor of extant acetylene hydratase may have functioned as enzyme for the oxidative addition of H_2_O to acetylene to generate acetyl thioester and glycolate. As evidenced here, these reactions could have proceeded still earlier non-enzymatically in volcanic-hydrothermal vent scenarios. Carbon monoxide is found in volcanic exhalations. At low temperatures, the equilibrium CO:CO_2_ molar ratio is low, but higher molar equilibrium ratios at high temperatures and pressures, e.g., 1:1 at 1200, °C and 2000, bar [29], could be conserved downstream by quenching [30]. Therefore, a mixture of acetylene and CO can be seen as a geochemically plausible carbon source for the synthesis of biomolecules under primordial conditions. Iron and nickel are the most abundant transition metals in the crust of the Earth [31], and iron–nickel sulfides are formed at the early stages of crustal evolution [32]. Nickel and iron–nickel centers are still widely spread in extant enzymes and catalyze a variety of reactions [33]. The here-described reactions showed a clear preference to NiS as catalyst, but for evolving further reaction cascades, e.g., reductive amination [34], a mixed FeS/NiS catalyst may be advantageous.

**Figure 2:**
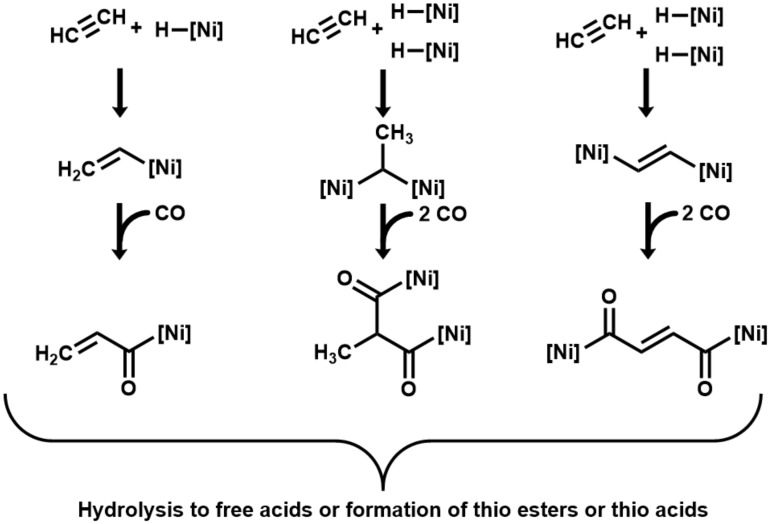
Proposed mechanism of acetylene/carboxydotrophic reactions on catalytic nickel centers. ([Ni] signifies a catalytic nickel center).

As initial interaction in the here-investigated acetylene/CO/NiS system, we suggest the coordination of acetylene and CO as ligands to Ni centers. The oxidation of the CO ligand to CO_2_ would generate hydride ligands. As next stages, we propose end-on organo–metal adducts between acetylene and one or two Ni centers, hydride transfer, and carbonyl insertion to form highly energetic acyl-[Ni] intermediates [35], which may hydrolyze to carboxylic acids with a total loss of the organo–metal energy. Instead of hydrolysis to free acids, they may react with a mercaptan (or H_2_S) to form thioesters (or thioacids) with partial energy conservation (Figure 2). The unsaturated carboxylates (acrylate and fumarate) that result from hydrolysis may subsequently convert by hydrogenation with CO as reductant to propionate and succinate. The addition of H_2_O may lead to the formation of lactate and malate.

In the context of a volcanic hydrothermal flow setting, the continuous supply of starting materials permits a metabolism with linear carbon fixation pathways that co-radiate from the ligand sphere of NiS. The products of these radial, linear pathways would have operated as pre-established stepping stones for later piecemeal cyclization. Subsequently, a scarcity of starting materials would have been compensated for by a conversion to autocatalytic CO_2_ fixation cycles, involving nutrient replacement, energy coupling, enzymatization, and the replacement of organo–metal activation by thioester activation without the violation of the principle of continuity. In our opinion, all extant carbon fixation cycles could be seen as successors of this primordial linear reaction system.

## Figures and Tables

**Table 1:** Metabolic products of the nickel-catalyzed reaction of acetylene with carbon monoxide. Reactions were carried out in 125 mL serum bottles with 5 mL of aqueous liquid phase for 7 days at 105 °C; products were identified by GC–MS as tert-butyldimethylsilyl derivatives. n.d.: not detected.

Runs		A	B	C	D
**NiSO_4_ • 6 H_2_O (mmol)**		1.0	0.5	-	0.5
**FeSO_4_ • 7 H_2_O (mmol)**		-	-	-	0.5
**β-Ni(OH)_2_ (mmol)**		-	0.5	1.0	-
**Na_2_SO_4_ (mmol)**		-	0.5	1.0	-
**Na_2_S • 9 H_2_O (mmol)**		1.0	0.5	-	1.0
**NaOH (mmol)**		0.5	0.5	0.5	0.5
**CO (ml)**		60	60	60	60
**C_2_H_2_ (ml)**		60	60	60	60
**pH end**		8.0	8.1	9.8	8.5
**Products (µM)**	**Chemical formula**				
**C1**					
**formate**	HCOO^-^	**18983**	**24207**	**310**	**434**
**C2**					
**acetate**	CH_3_COO^-^	**4358**	**3434**	**112**	**749**
**glycolate**	HOCH_2_COO^-^	**32**	**38**	**n.d.**	**11**
**C3**					
**acrylate**	CH_2_CHCOO^-^	**9692**	**16874**	**243**	**763**
**propionate**	CH_3_CH_2_COO^-^	**10368**	**15021**	**171**	**339**
**pyruvate**	CH_3_COCOO^-^	**43**	**117**	**n.d.**	**4**
**β-lactate**	HOCH_2_CH_2_COO^-^	**273**	**793**	**n.d.**	**n.d.**
**glycerate**	HOCH_2_CH_2_(OH)COO^-^	**108**	**102**	**n.d.**	**n.d.**
**C4**					
**crotonate**	CH_3_CHCHCOO^-^	**226**	**516**	**n.d.**	**22**
**2-methylmalonate**	^-^OOCCH(CH_3_)COO^-^	**48**	**145**	**n.d.**	**n.d.**
**maleate**	^-^OOCCHCHCOO^-^	**72**	**585**	**n.d.**	**14**
**succinate**	^-^OOCCH_2_CH_2_COO^-^	**3964**	**4747**	**3**	**187**
**fumarate**	^-^OOCCHCHCOO^-^	**358**	**391**	**n.d.**	**12**
**malate**	^-^OOCCH(OH)CH_2_COO^-^	**17**	**85**	**n.d.**	**n.d.**
**C5**					
**(E)-2-methylbut-2-enoate**	CH_3_CHC(CH_3_)COO^-^	**196**	**411**	**n.d.**	**n.d.**
**∑ C2–C5**		**29755**	**43259**	**358**	**2101**

**Table 2:** Formation of methyl thioacetate (thioacetic acid S-methyl ester; CH_3_COSCH_3_) from acetylene and methane thiol with or without carbon monoxide. Reactions were carried out in 125 ml serum bottles with 10 ml of aqueous liquid phase for 1 day at 105 °C; methyl thioacetate was identified by GC–MS after ethyl acetate extraction. Labelling in characteristic fragments is shown for runs with D_2_O or ^13^CO. n^ signifies D-labels, n• signifies n ^13^C-labels.

Runs	A	B	Labelling in Characteristic Fragments	
			Mass 1	Mass 2
NiSO_4_ • 6 H_2_O (mmol)	2	2		
Na_2_S • 9 H_2_O (mmol)	1.5	1.5		
NaOH (mmol)	0.6	0.6		
C_2_H_2_ (ml)	90	45		
CO (ml)	-	45		
CH_3_SH (ml)	25	25		
**Methyl thioacetate (µM)**	**2**	**4**	90_3^0•	43_3^0•

**Table 3:** Consecutive products from selected acids in the presence of CO. Reactions were carried out in 125 mL serum bottles with 5 mL of aqueous liquid phase and 120 ml of CO as gaseous phase for 7 days at 105 °C; further conditions are as described in run A of Table 1, replacing acetylene by 0.5 mmol of the indicated substrates. Products were identified by GC–MS as tert-butyldimethylsilyl derivatives.

Runs		D	E	F	G
**Substrate**		fumarate	malate	acrylate	succinate
**Product (%)**	**Chemical formula**				
**acrylate**	CH_2_CHCOO^-^	**n.d.**	**n.d.**	**6.37**	**n.d.**
**propionate**	CH_3_CH_2_COO^-^	**n.d.**	**n.d.**	**6.80**	**n.d.**
**β-lactate**	HOCH_2_CH_2_COO^-^	**n.d.**	**n.d.**	**39.21**	**n.d.**
**maleate**	^-^OOCCHCHCOO^-^	**2.57**	**0.02**	**0.31**	**n.d.**
**succinate**	^-^OOCCH_2_CH_2_COO^-^	**71.67**	**1.13**	**43.83**	**99.82**
**fumarate**	^-^OOCCHCHCOO^-^	**21.94**	**0.24**	**1.14**	**0.14**
**malate**	^-^OOCCH(OH)CH_2_COO^-^	**3.70**	**98.58**	**2.36**	**0.04**

## Supplementary Materials

The following are available online at https://www.mdpi.com/2075-1729/10/4/35/s1. Table S1. Metabolic products of the nickel-catalyzed reaction of acetylene with carbon monoxide. Figure S1. Formation of methyl thioacetate (thioacetic acid S-methyl ester) from HC≡CH and CH3SH in the presence of NiS. Figure S2. Comparison of the reductive acetyl-CoA pathway and the proposed primordial reaction mechanism to thioacetate. Figure S3. Reductive tricarboxylic acid cycle. Figure S4. 3-Hydroxpropinate/4-hydroxybutyrate cycle. Figure S5. Dicarboxylate/4-hydroxbutyrate cycle. Figure S6. 3-Hydroxypropionate bicycle.
