# Supplementary material for: A Possible Primordial Acetyleno/Carboxydotrophic Core Metabolism
Source: Life (Basel). 2020 Apr 7;10(4):35. doi: 10.3390/life10040035 (PMC7235995; doi:10.3390/life10040035)
Supplement: Supplementary file 1 [file life-10-00035-s001.zip › Supplementary Information Huber 02_04_2020.pdf]

Supplementary Information

A Possible Primordial Acetyleno/Carboxydotrophic Core Metabolism

Jessica Sobotta[†], Thomas Geisberger[†], Carolin Moosmann, Christopher M. Scheidler, Wolfgang Eisenreich, Günter Wächtershäuser, Claudia Huber[*]

Table S1: Metabolic products of the nickel catalyzed reaction of acetylene with carbon monoxide

Figure S1: Formation of methyl thioacetate (thioacetic acid S-methyl ester) from HC≡CH and CH_3SH in the presence of NiS

Figure S2: Comparison of the Reductive acetyl-CoA pathway4 and the proposed primordial reaction mechanism to thio acetate

Figure S3: Reductive tricarboxylic acid cycle

Figure S4: 3-Hydroxpropinate/4-hydroxybutyrate cycle

Figure S5: Dicarboxylate/4-Hydroxbutyrate cycle

Figure S6: 3-Hydroxypropionate bicycle

Additional references: [39-44]

Table S1: Metabolic products of the nickel catalyzed reaction of acetylene with carbon monoxide. Reactions were carried out in 125 ml serum bottles with 5 ml aqueous liquid phase for 7 days at 105 °C; Products were identified by GC-MS as *tert*-butyldimethylsilyl derivatives; Labelling in characteristic fragments is shown for runs with D_2O or ^{13}CO. n^ signifies n D-labels, n• signifies n ^{13}C-labels, 0.5• signifies 50% labelling of the indicated product.

**Runs**	**A**	**B**	**C**	**D**			
$NiSO_4$ • 6 H_2O [mmol]	1	0.5		0.5			
$FeSO_4$ • 7 H_2O [mmol]				0.5			
β-$Ni(OH)_2$ [mmol]	-	0.5	1	-			
Na_2SO_4 [mmol]	-	0.5	1	-			
Na_2S • 9 H_2O [mmol]	1	0.5	-	1.0			
NaOH [mmol]	0.5	0.5	0.5	0.5			
CO [ml]	60	60	60	60			
C_2H_2 [ml]	60	60	60	60			
pH_{end}	8.0	8.1	9.8	8.5			
**Products [μM]**					labelling in characteristic fragments		
					mass1	mass2	mass3
**C1**							
formate	**18983**	**24207**	**310**	**434**	145_1^1•	103_1^1•	
**C2**							
acetate	**4358**	**3434**	**112**	**749**	117_3^0•	99_0^0•	75_1^0•
glycolate	**32**	**38**	**n.d.**	**11**	247_2^0•	219_2^0•	163_2^0•
**C3**							
acrylate	**9692**	**16874**	**243**	**763**	129_3^1•	99_1^1•	85_3^0•
propionate	**10368**	**15021**	**171**	**339**	131_5^1•	115_0^0•	173_5^1•
pyruvate	**43**	**117**	**n.d.**	**4**	259_2^0.5•	231_0^0•	189_0^0•
β-lactate	**273**	**793**	**n.d.**	**n.d.**	261_4^1•	219_3^0•	163_2^0•
glycerate	**108**	**102**	**n.d.**	**n.d.**	391_3^0.5•	363_3^0•	289_3^0•
**C4**							
but-3-enoate	**187**	**563**	**n.d.**	**n.d.**	143_5^1•	115_0^0•	99_5^0•
crotonate	**226**	**516**	**n.d.**	**22**	143_5^0.5•	99_5^0•	185_0^1•
2-methylmalonate	**48**	**145**	**n.d.**	**n.d.**	289_3^2•	331_0^2•	133_0^0•
maleate	**72**	**585**	**n.d.**	**14**	287_2^2•	329_2^2•	115_0^1•
succinate	**3964**	**4747**	**3**	**187**	289_4^2•	331_4^2•	215_4^2•
fumarate	**358**	**391**	**n.d.**	**12**	287_2^2•	245_2^2•	329_2^2•
2,3-dihydroxybutyrate	**31**	**41**	**n.d.**	**n.d.**	405_5^1•	377_5^0•	303_5^0•
malate	**17**	**85**	**n.d.**	**n.d.**	419_3^2•	287_2^2•	461_3^0•
**C5**							
(E)-2-methylbut-2-enoate	**196**	**411**	**n.d.**	**n.d.**	157_7^1•	113_7^0•	83_7^1•

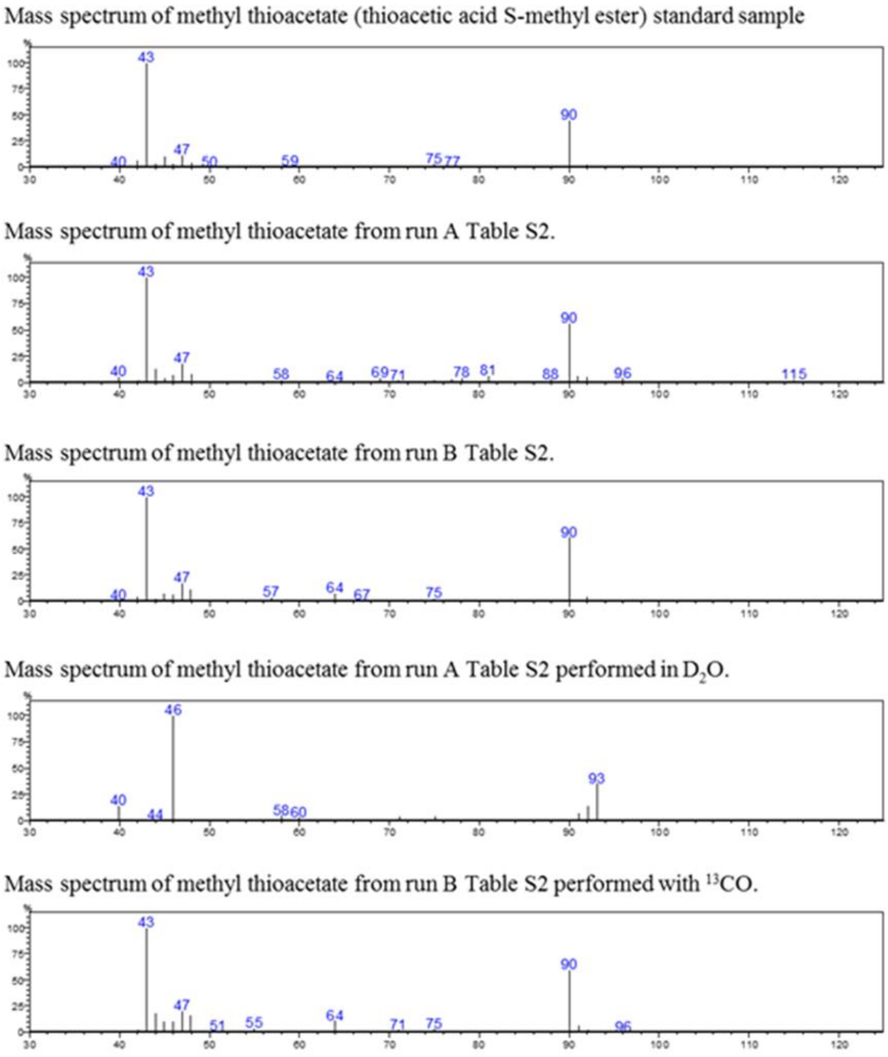

Figure S1: Formation of methyl thioacetate (thioacetic acid S-methyl ester) from HC≡CH and CH_3SH in the presence of NiS. Mass spectra are shown for the acetyl thioester standard sample as well as for experiments with HC≡CH (Table 3 run A), HC≡CH + CO (Table 3 run B), D_2O and ^{13}CO.

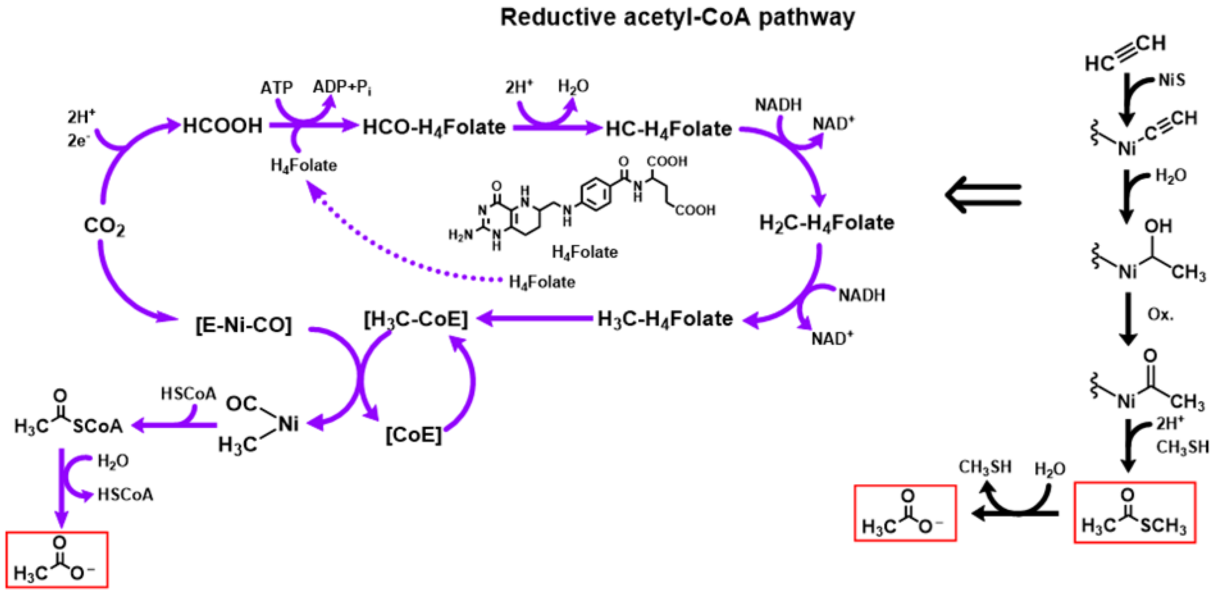

Figure S2: Comparison of the Reductive acetyl-CoA pathway[11] and the proposed primordial reaction mechanism to thio acetate. Metabolites highlighted by red boxes correspond to products of the NiS/HC≡CH/CO system. Pathway is adapted from[11,39]. CoE stands for corrinoid Enzyme, H_3C-CoE for methyl corrinoid protein; E-Ni-CO for CO dehydrogenase complex.

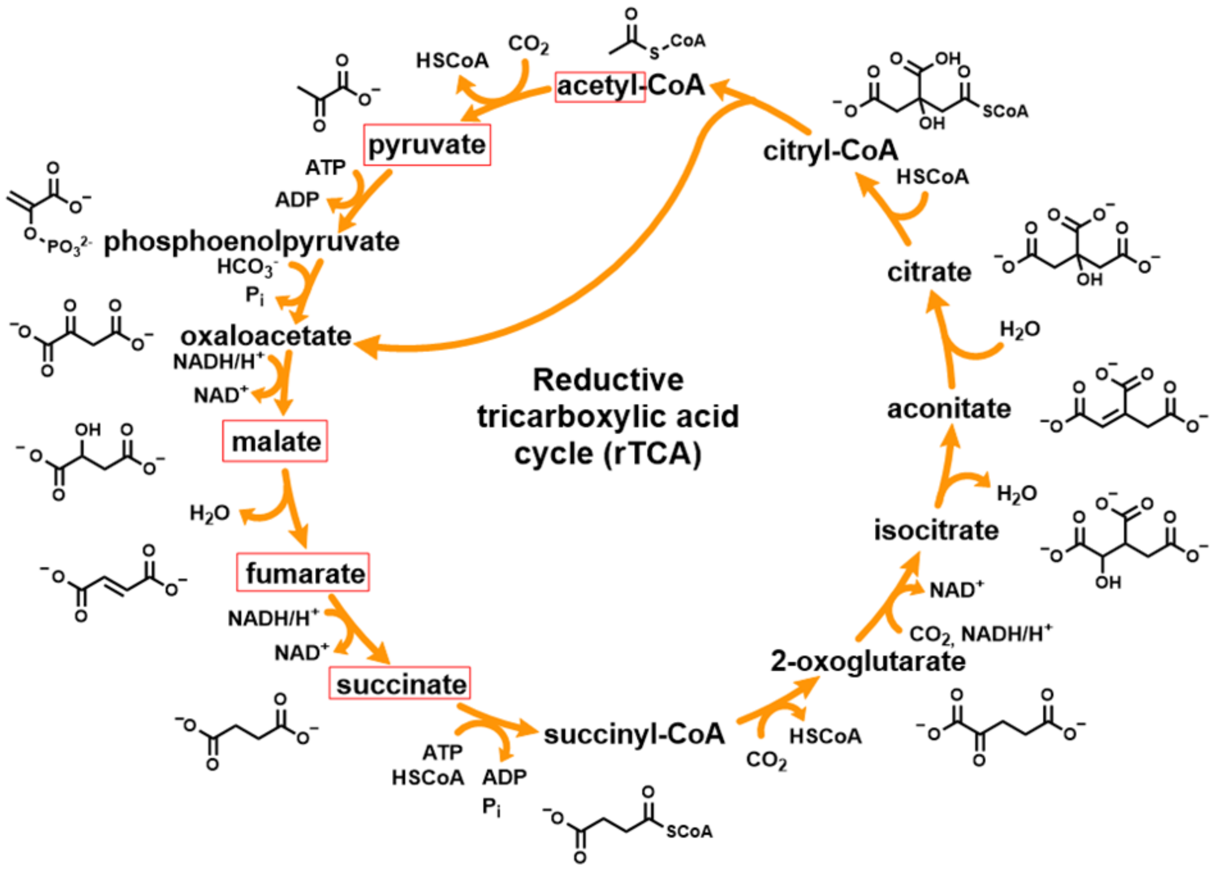

Figure S3: Reductive tricarboxylic acid cycle[14]. Metabolites highlighted by red boxes correspond to products of the NiS/HC≡CH/CO system. Graphics partly adapted from[40,41]

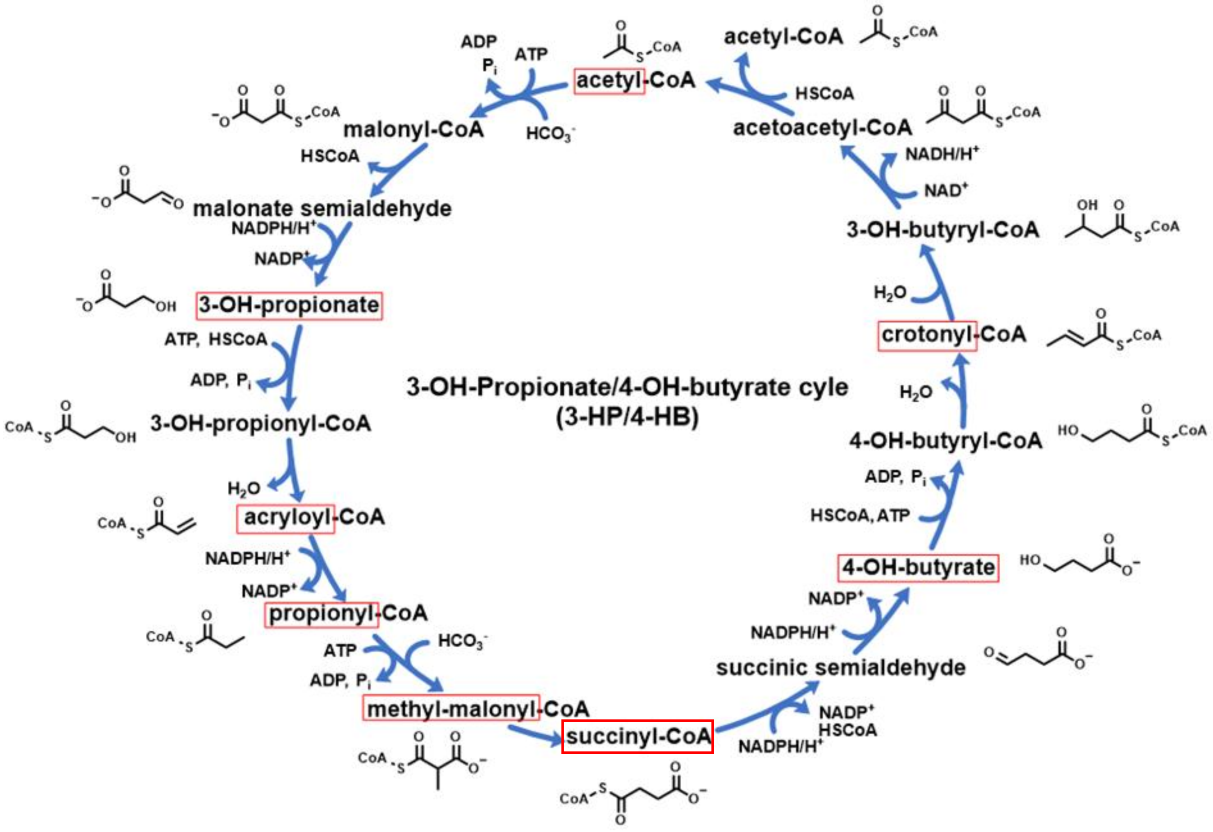

Figure S4: 3-Hydroxpropinate/4-hydroxybutyrate cycle[15]. Metabolites highlighted by red boxes correspond to products of the NiS/HC≡CH/CO system. Graphics partly adapted from[15,42].

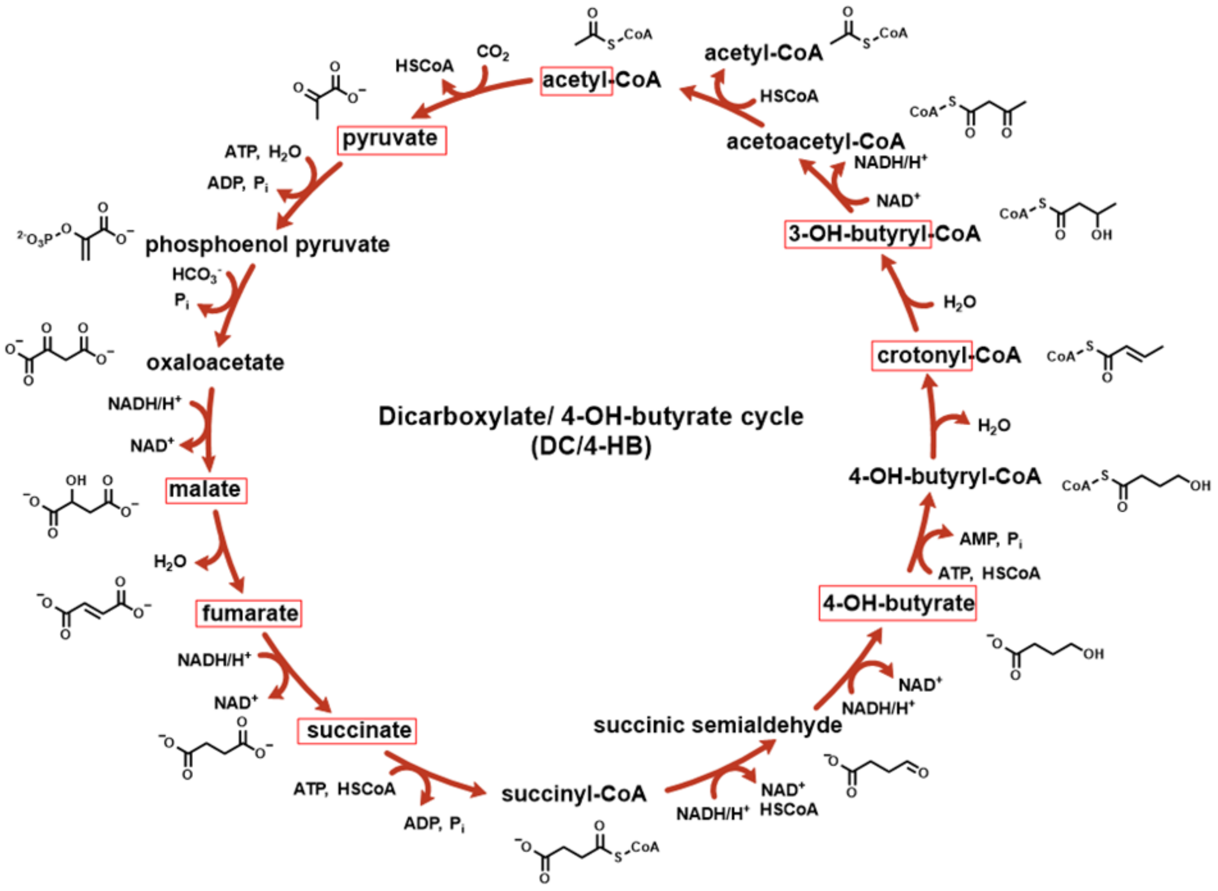

Figure S5: Dicarboxylate/4-Hydroxbutyrate cycle[16]. Metabolites highlighted by red boxes correspond to products of the NiS/HC≡CH/CO system. Graphics partly adapted from[16,43].

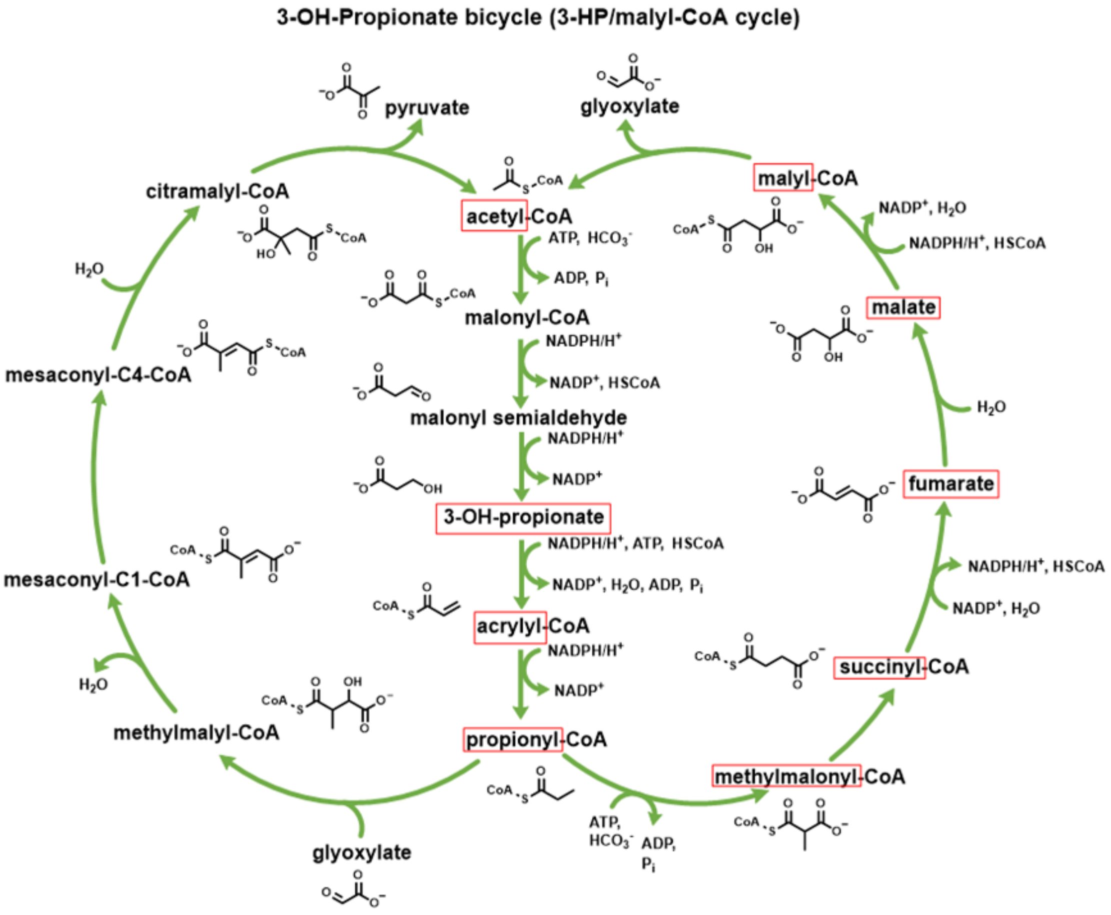

Figure S6: 3-Hydroxypropionate bicycle[17]. Metabolites highlighted by red boxes correspond to products of the NiS/HC≡CH/CO system. Graphics partly adapted from[17,44].

Additional references

39. Ljungdahl, L. G. The Autotrophic Pathway of Acetate Synthesis in Aceto Genic Bacteria. *Ann. Rev. Microbiol.* **1986**, *40*, 415-50.
40. Kitadai, N.; Kameya, M.; Fujishima, K. Origin of the reductive tricarboxylic acid (rtca) cycle-type CO_2 fixation: A perspective. *Life (Basel)* **2017**, *7*, 16.
41. Wahlund, T. M.; Tabita, F. R. The Reductive Tricarboxylic Acid Cycle of Carbon Dioxide Assimilation: Initial Studies and Purification of ATP-Citrate Lyase from the Green Sulfur Bacterium *Chlorobium tepidum*. *J Bacteriol* **1997**, *179*, 4859-4867.
42. Tully, B. J., Nelson, W. C.; Heidelberg, J. F. Metagenomic analysis of a complex marine planktonic thaumarchaeal community from the Gulf of Maine. *Environ. Microbiol.* **2012**, *14*, 254–267.
43. Berg, I. A.; Kockelkorn D.; Ramos-Vera W. H.; Say R. F.; J. Zarzycki; Hügler M.; Alber B. E.; Fuchs G. Autotrophic carbon fixation in archaea. *Nat. Rev. Microbiol.* **2010**, *8*, 447-460.
44. Zarzycki, J., Brecht, V., Müller, M.; Fuchs, G. Identifying the missing steps of the autotrophic 3-hydroxypropionate CO_2 fixation cycle in *Chloroflexus aurantiacus*. *PNAS* **2009**, *106*, 21317-21322.
